# Association Between Daily Energy Intake and Cognitive Recovery After Mechanical Thrombectomy for Acute Ischemic Stroke: A Multicenter Retrospective Study

**DOI:** 10.3390/jcm15218333

**Published:** 2026-10-26

**Authors:** Sergiu Florin Arnăutu, Diana Aurora Arnăutu, Tufail Patankar, Mohd Shariq, Hamood Qazi, Martin Lewis, Sara Sciacca, Dragoș Cătălin Jianu, Octavian M. Crețu, Dorel Sandesc, Claudiu-Daniel Malița, Cătălin Juratu, Brenda-Cristiana Bernad, Jeremy Lynch

**Affiliations:** 1 Centre for Cognitive Research in Neuropsychiatric Pathology (Neuro-Psy-Cog), Department of Neurosciences, “Victor Babes” University of Medicine and Pharmacy from Timisoara, E. Murgu Sq., No. 2, 300041 Timisoara, Romania; arnautu.sergiu@umft.ro (S.F.A.);; 2 Department of Internal Medicine, “Victor Babes” University of Medicine and Pharmacy from Timisoara, E. Murgu Sq., No. 2, 300041 Timisoara, Romania; 3 Department of Interventional Neuroradiology, Queen’s Hospital, Barking, Havering and Redbridge University Hospitals NHS Trust, London RM7 0AG, UK; 4 Department of Biomedical Imaging Science, Leeds Institute of Cardiovascular and Metabolic Medicine, University of Leeds, Clarendon Way, Leeds LS2 9JT, UK; 5 Department of Stroke Medicine, Queen’s Hospital, Barking, Havering and Redbridge University Hospitals NHS Trust, London RM7 0AG, UK; 6 Department of Neurosciences, King’s College Hospital NHS Foundation Trust, London SE5 9RS, UK; 7 First Division of Neurology, Department of Neurosciences, “Victor Babes” University of Medicine and Pharmacy from Timisoara, Eftimie Murgu Sq. No. 2, 300041 Timisoara, Romania; 8 Department of Neurology I, Pius Brînzeu County Clinical Emergency Hospital, 156 L. Rebreanu Ave., 300723 Timisoara, Romania; 9 First Department of Surgery—Clinic of Surgical Semiotics and Thoracic Surgery, Centre for Hepato-Biliary and Pancreatic Surgery, “Victor Babes” University of Medicine and Pharmacy from Timisoara, Eftimie Murgu Sq. No. 2, 300041 Timisoara, Romania; 10 Department of Anesthesia and Intensive Care, “Victor Babes” University of Medicine and Pharmacy from Timisoara, E. Murgu Sq., No. 2, 300041 Timisoara, Romania; 11 Department of Radiology, “Victor Babes” University of Medicine and Pharmacy from Timisoara, E. Murgu Sq., No. 2, 300041 Timisoara, Romania; 12 Department of Interventional Radiology, Pius Brînzeu County Clinical Emergency Hospital, 156 L. Rebreanu Ave., 300723 Timisoara, Romania; 13 Doctoral School, “Victor Babes” University of Medicine and Pharmacy from Timisoara, E. Murgu Sq., No. 2, 300041 Timisoara, Romania; 14 Department of Neuroscience, “Victor Babes” University of Medicine and Pharmacy from Timisoara, E. Murgu Sq., No. 2, 300041 Timisoara, Romania; 15 Centre for Neuropsychology and Behavioural Medicine, “Victor Babes” University of Medicine and Pharmacy from Timisoara, Eftimie Murgu Sq. No. 2, 300041 Timisoara, Romania

**Keywords:** acute ischemic stroke, mechanical thrombectomy, cognitive recovery, Montreal Cognitive Assessment, daily energy intake, nutrition, rehabilitation

## Abstract

**Background**: Post-stroke cognitive impairment is a common complication associated with reduced functional independence and quality of life. Although adequate daily energy intake is considered an essential component of stroke rehabilitation, its association with cognitive recovery after mechanical thrombectomy remains poorly understood. This study investigated the relationship between daily energy intake and 90-day cognitive outcomes in patients with acute ischemic stroke treated with mechanical thrombectomy. **Methods**: This multicenter retrospective observational cohort study included 100 consecutive patients undergoing mechanical thrombectomy between 1 April 2025 and 1 April 2026 at stroke centers in the United Kingdom and Romania. Daily energy and macronutrient intake were retrospectively extracted from routine clinical records and normalized to body weight (kcal/kg/day). Daily energy intake was evaluated primarily as a continuous exposure (kcal/kg/day), while the originally defined 31 kcal/kg/day grouping was retained for exploratory categorical analyses. Cognitive performance was assessed using the Montreal Cognitive Assessment (MoCA) at baseline and, among survivors with available cognitive follow-up, at 90 days. The primary multivariable model evaluated daily energy intake (kcal/kg/day) as a continuous exposure and adjusted for baseline MoCA, atrial fibrillation, chronic coronary artery disease, admission NIHSS, C-reactive protein, and PICA/AICA/SCA occlusion. HC3 heteroscedasticity-consistent standard errors were used for robust inference. **Results**: Among patients with available 90-day cognitive assessment, those with higher energy intake achieved significantly higher 90-day MoCA scores than those with lower intake (24.2 ± 2.1 vs. 20.0 ± 1.5; *p* < 0.001) and greater cognitive improvement (ΔMoCA: 3.82 ± 1.08 vs. 0.48 ± 0.54 points; *p* < 0.001). In the primary continuous-exposure model, each additional 1 kcal/kg/day was associated with a 0.18-point higher 90-day MoCA score after multivariable adjustment (β = 0.179, HC3 95% CI 0.081–0.278; *p* < 0.001). The association remained similar in an expanded sensitivity model addressing available indicators of baseline severity (β = 0.176, HC3 95% CI 0.071–0.281; *p* = 0.002). Exploratory categorical analyses also showed higher 90-day MoCA scores in the higher-intake group. No significant differences were observed in NIHSS or mRS at 90 days. **Conclusions**: Higher daily energy intake was associated with higher 90-day cognitive performance among survivors with available cognitive follow-up after mechanical thrombectomy. Given the retrospective observational design, baseline differences between exposure groups, and potential residual confounding and reverse causality, these findings should be considered exploratory and hypothesis-generating. Prospective studies are required to determine whether nutritional optimization can influence cognitive recovery after stroke.

## 1. Introduction

Acute ischemic stroke is one of the leading causes of death and long-term disability worldwide [1]. Despite major advances in reperfusion therapies, including intravenous thrombolysis and mechanical thrombectomy, many survivors continue to experience persistent neurological deficits that adversely affect independence and quality of life. In recent years, mechanical thrombectomy has become the standard of care for patients with acute large-vessel occlusion, substantially improving functional outcomes compared with medical treatment alone. Nevertheless, successful recanalization does not always translate into complete neurological or cognitive recovery, highlighting the importance of identifying potentially modifiable factors that may influence rehabilitation and long-term outcomes [1,2,3,4,5].

Post-stroke cognitive impairment is a frequent complication affecting approximately one-third of stroke survivors and represents a major determinant of functional independence, medication adherence, return to work, and quality of life [6,7,8,9,10]. Recent systematic-review and meta-analytic evidence further demonstrates an association between post-stroke cognition and stroke-survivor quality of life and caregiver outcomes [11]. Cognitive deficits may involve attention, executive function, memory, language, and visuospatial abilities, even in patients with favorable neurological recovery. While neurological improvement is routinely assessed using the National Institutes of Health Stroke Scale (NIHSS) and global disability is commonly evaluated using the modified Rankin Scale (mRS), these instruments provide limited information regarding cognitive recovery. Consequently, formal cognitive assessment has become increasingly recognized as an important component of post-stroke follow-up [12,13]. A systematic review specifically examining cognitive outcomes after mechanical thrombectomy found substantial heterogeneity in cognitive assessment methods and available evidence, highlighting the need for further standardized investigation of post-thrombectomy cognitive outcomes [14].

Adequate nutritional support is an essential aspect of comprehensive stroke care. During the acute phase of ischemic stroke, patients frequently experience dysphagia, reduced oral intake, altered metabolic demands, and systemic inflammatory responses, all of which may contribute to negative energy balance and malnutrition [15,16,17,18,19,20]. Previous studies have shown that malnutrition after stroke is associated with increased mortality, prolonged hospitalization, poorer functional recovery, and higher rates of institutionalization. Nutritional status may also be relevant to cognitive outcome; in an observational cohort of patients with acute ischemic stroke, a lower Geriatric Nutritional Risk Index was associated with post-stroke cognitive impairment at 3 months [21]. A systematic review and meta-analysis has further demonstrated that impaired nutritional status is common across the post-stroke trajectory, from the hyperacute through the chronic phase [22]. Experimental evidence further suggests that adequate energy and protein intake may support neuroplasticity, neuronal repair, preservation of skeletal muscle mass, and participation in rehabilitation. Whether daily energy intake normalized to body weight is associated with 90-day cognitive performance after endovascular treatment therefore remains uncertain [15,23].

Most previous studies investigating nutrition after stroke have focused on malnutrition screening, nutritional supplementation, or functional outcomes. Comparatively few studies have evaluated whether the amount of daily energy intake is associated with cognitive performance following mechanical thrombectomy. Moreover, the majority of available evidence has assessed total caloric intake without normalization to body weight, despite current nutritional recommendations commonly expressing energy requirements in kcal/kg/day. Whether higher energy intake relative to body weight is independently associated with improved cognitive recovery after successful endovascular treatment therefore remains uncertain [15,23].

The present multicenter retrospective observational cohort study aimed to investigate the association between daily energy intake and cognitive recovery in patients with acute ischemic stroke treated with mechanical thrombectomy. Daily energy intake was evaluated both as absolute energy intake (kcal/day) and normalized to body weight (kcal/kg/day). Cognitive recovery was assessed using the Montreal Cognitive Assessment (MoCA) at baseline and 90-day follow-up, while neurological and functional outcomes were evaluated using the NIHSS and modified Rankin Scale. We hypothesized that higher daily energy intake would be associated with higher 90-day cognitive performance after accounting for measured baseline characteristics.

## 2. Materials and Methods

### 2.1. Study Design and Setting

This multicenter retrospective observational cohort study included consecutive adult patients with acute ischemic stroke who underwent mechanical thrombectomy between 1 April 2025 and 1 April 2026.

Patients were identified from three comprehensive stroke centers:

Queen’s Hospital, Barking, Havering and Redbridge University Hospitals NHS Trust, London, United Kingdom;

Timișoara County Emergency Clinical Hospital, Timișoara, Romania;

Timișoara Municipal Emergency Clinical Hospital, Timișoara, Romania.

Clinical, imaging, nutritional, laboratory, neurological, functional, and cognitive data were retrospectively extracted from electronic medical records and hospital databases.

The study was conducted in accordance with the Declaration of Helsinki. Ethical approval for the Romanian cohort was obtained from the Ethics Committee of the Municipal Clinical Emergency Hospital, Timișoara (Approval No. I-29417/9 November 2022). Data from Queen’s Hospital were retrospectively obtained from anonymized routinely collected clinical records; in accordance with applicable local NHS governance requirements, additional Research Ethics Committee approval was not required for this analysis.

### 2.2. Study Population

All consecutive adult patients undergoing mechanical thrombectomy during the study period were screened for eligibility. Patients were eligible if they were aged ≥18 years, had an acute ischemic stroke confirmed by CT and/or MRI with a large-vessel occlusion treated by mechanical thrombectomy, and had sufficient clinical documentation for the planned analyses. Required data included body weight and body mass index, nutritional records permitting calculation of daily energy intake (kcal/day and kcal/kg/day) and macronutrient intake, admission NIHSS, pre-stroke mRS, baseline MoCA, and available follow-up outcome data.

Patients were excluded if they had previously diagnosed dementia or another major neurocognitive disorder, severe aphasia or persistent impairment of consciousness precluding reliable cognitive assessment, acute delirium at the time of MoCA testing, severe psychiatric disease interfering with cognitive assessment, or another neurological disorder associated with substantial pre-existing cognitive impairment. Patients with insufficient nutritional records, unavailable body weight, or unavailable cognitive assessment required for the respective cognitive analyses were also excluded. Patients who died before the 90-day cognitive assessment were retained in analyses of mortality and functional outcome but were not included in analyses requiring a 90-day MoCA score.

### 2.3. Clinical and Imaging Assessment

All patients underwent mechanical thrombectomy according to institutional protocols and contemporary international recommendations for endovascular treatment of acute ischemic stroke.

Patient selection for thrombectomy was based on multidisciplinary evaluation incorporating clinical presentation, neurological examination, non-contrast CT, CT angiography and, where appropriate, CT perfusion or MRI.

Mechanical thrombectomy was performed by experienced interventional neuroradiologists using contemporary aspiration catheters, stent retrievers, balloon-guide catheters, or combined techniques according to operator preference and vascular anatomy [1].

Intravenous thrombolysis was administered before thrombectomy whenever indicated and not contraindicated.

Post-procedural management was performed in dedicated stroke units according to local institutional protocols.

### 2.4. Nutritional Assessment

Nutritional data were retrospectively extracted from routinely documented electronic medical records and clinical documentation recorded during the index hospitalization. The collected nutritional variables included daily energy intake (kcal/day), daily protein intake (g/day), daily carbohydrate intake (g/day), and daily lipid intake (g/day), as documented as part of routine inpatient care.

Body weight measured during hospitalization was used to calculate daily energy intake normalized to body weight (kcal/kg/day) according to the following formula:

kcal/kg/day = daily energy intake (kcal/day) ÷ body weight (kg).

To assess the internal consistency of the nutritional data, the recorded daily energy intake was verified using the Atwater conversion factors (4 kcal/g for protein, 4 kcal/g for carbohydrate, and 9 kcal/g for lipid). No modifications were made to the original nutritional data extracted from the medical records.

Daily energy intake was evaluated primarily as a continuous exposure (kcal/kg/day). For exploratory categorical analyses, the previously defined 31 kcal/kg/day grouping was retained, classifying patients as lower (<31 kcal/kg/day) or higher (≥31 kcal/kg/day) energy intake. In the revised ROC analysis restricted to survivors with available 90-day MoCA assessment, the within-sample Youden threshold was estimated separately and was not used to redefine the exposure groups.

### 2.5. Data Collection

Baseline demographic and anthropometric variables included age, sex, body mass index, and body weight. Vascular risk factors included hypertension, atrial fibrillation, dyslipidemia, and chronic coronary artery disease. Medication data included the use of angiotensin-converting enzyme inhibitors, calcium-channel blockers, β-blockers, antiplatelet agents, statins, and antibiotic treatment during hospitalization. Laboratory variables included serum albumin and C-reactive protein (CRP). Stroke-related variables included the initial imaging modality and arterial occlusion site, categorized as tandem, internal carotid artery terminus, M1, M2 inferior division, M2 superior division, basilar artery, or PICA/AICA/SCA territory.

### 2.6. Outcomes

Stroke severity was assessed using the National Institutes of Health Stroke Scale (NIHSS) at admission, hospital discharge, and 90-day follow-up [24].

Functional outcome was evaluated using the modified Rankin Scale (mRS) before stroke and at 90 days [25].

An mRS score of 6 represented death. Ninety-day mortality was therefore derived directly from the 90-day mRS assessment.

Cognitive performance was assessed using the Montreal Cognitive Assessment (MoCA). Baseline MoCA was performed during the index hospitalization after neurological stabilization and before hospital discharge, whereas follow-up assessment was performed approximately 90 days after stroke. Patients with severe aphasia, persistent disorders of consciousness, or other conditions precluding reliable cognitive assessment were excluded from analyses involving MoCA [12].

The primary cognitive endpoint was the 90-day MoCA score among patients alive at 90 days with an available cognitive assessment.

Secondary endpoints included the following:

change in MoCA (ΔMoCA);
NIHSS at discharge;
NIHSS at 90 days;
90-day functional outcome;
90-day mortality.

### 2.7. Statistical Analysis

Statistical analyses were performed using R version 4.6.1 (2026-06-24 ucrt) in RStudio version 2026.07.1 (Build 147). All statistical tests were two-sided, and a *p*-value < 0.05 was considered statistically significant.

Daily energy intake normalized to body weight (kcal/kg/day) was evaluated as a continuous exposure to avoid reliance on data-driven categorization. For exploratory categorical analyses, patients were additionally classified into lower (<31 kcal/kg/day) and higher (≥31 kcal/kg/day) energy-intake groups using the internally derived threshold described above. Continuous variables were summarized as mean ± standard deviation (SD). Ordinal and non-normally distributed variables, including the National Institutes of Health Stroke Scale (NIHSS) and C-reactive protein (CRP), were summarized as median [interquartile range (IQR)]. Categorical variables were reported as number and percentage.

Because pre-stroke and 90-day modified Rankin Scale (mRS) scores were concentrated toward the lower end of the scale, these outcomes were summarized in Table 1 as the proportion of patients with mRS > 0. The full 90-day mRS distribution was additionally presented graphically.

Unadjusted comparisons between the low and higher energy-intake groups were performed using Welch’s two-sample *t*-test for continuous variables, the Wilcoxon rank-sum test for ordinal or skewed variables, and Pearson’s chi-square test or Fisher’s exact test, as appropriate, for categorical variables. These methods correspond to those used to generate the final comparative Table 1.

Baseline characteristics, 90-day mRS, and mortality were analyzed in the overall cohort. Because mRS 6 represents death, deceased patients remained included in functional-outcome and mortality analyses. Analyses involving 90-day MoCA and ΔMoCA were restricted to patients alive at 90 days with available cognitive follow-up. Cognitive outcomes were not imputed for deceased patients, and complete-case analysis was used for analyses requiring 90-day MoCA. The group denominators of 50 refer to the overall cohort. Twelve patients died before the 90-day follow-up (mRS 6: 9 in the lower energy-intake group and 3 in the higher energy-intake group). Cognitive outcomes at 90 days were calculated only among surviving patients with available MoCA assessments; deceased patients were not assigned or imputed cognitive scores.

The primary cognitive outcome was the Montreal Cognitive Assessment (MoCA) score at 90 days. Because the 31 kcal/kg/day threshold was derived within the study cohort, the principal adjusted analysis treated daily energy intake normalized to body weight as a continuous exposure. Multivariable linear regression within an analysis-of-covariance framework was performed with 90-day MoCA as the dependent variable and daily energy intake (kcal/kg/day) as the principal exposure. The model was adjusted for baseline MoCA, atrial fibrillation, chronic coronary artery disease, NIHSS at admission, C-reactive protein (CRP), and PICA/AICA/SCA occlusion. HC3 heteroscedasticity-consistent standard errors were used for inference. Results are reported as adjusted regression coefficients (β) with 95% confidence intervals (CI) and *p*-values. The higher-versus-lower energy-intake comparison based on the internally derived 31 kcal/kg/day threshold was retained as an exploratory categorical analysis rather than the primary inferential model.

Change in cognitive performance was calculated as follows:

ΔMoCA = 90-day MoCA − baseline MoCA. ΔMoCA was calculated only for patients with both an index-hospitalization baseline MoCA and an available 90-day MoCA assessment.

Positive ΔMoCA values indicated cognitive improvement. The between-group difference in ΔMoCA was evaluated using Welch’s two-sample *t*-test. Neurological change was calculated as 90-day NIHSS minus admission NIHSS, with negative values indicating neurological improvement, and was compared using the Wilcoxon rank-sum test.

As sensitivity analyses, the primary continuous-exposure model was expanded to include additional available indicators of baseline clinical status, including age, sex, and pre-stroke mRS. A separate analysis was also performed after excluding patients with PICA/AICA/SCA occlusion to examine whether the association persisted in the absence of the most markedly imbalanced occlusion category. These analyses were intended to assess robustness to measured baseline differences and do not eliminate the possibility of residual or unmeasured confounding.

Propensity-score matching (PSM) was additionally performed as a sensitivity analysis to assess whether the marked baseline differences between the exploratory energy-intake groups could be adequately balanced. The propensity-score model included age, sex, BMI, hypertension, atrial fibrillation, dyslipidaemia, chronic coronary artery disease, imaging modality, admission NIHSS, pre-stroke mRS, baseline MoCA, and PICA/AICA/SCA occlusion. One-to-one nearest-neighbor matching without replacement was attempted using a caliper of 0.20 standard deviations of the propensity-score logit. Covariate balance was assessed using standardized mean differences (SMDs), with an absolute SMD < 0.10 considered indicative of adequate balance. Because the two exposure groups demonstrated limited propensity-score overlap and substantial residual imbalance after conventional matching, particularly for atrial fibrillation and PICA/AICA/SCA occlusion, the matched estimates were considered exploratory and were not used as the principal effect estimates. Exact matching on PICA/AICA/SCA status was additionally explored, but substantial residual imbalance in other baseline covariates persisted.

No formal a priori sample-size calculation was performed because this retrospective study included all consecutive eligible patients during the predefined study period. To characterize the statistical sensitivity of the available cognitive-analysis sample, a minimum detectable effect-size analysis was performed for the seven-predictor primary regression model. With 88 patients, seven predictors, a two-sided α of 0.05, and 80% power, the minimum detectable Cohen’s f^2^ was approximately 0.178; consequently, smaller associations may not have been detectable with the available sample. Model assumptions were examined using residual diagnostic plots. Multicollinearity was assessed using variance inflation factors, heteroscedasticity using the Breusch–Pagan test, residual distribution using the Shapiro–Wilk test, and influential observations using Cook’s distance. Because deviations from conventional regression assumptions could affect standard errors, HC3 heteroscedasticity-consistent standard errors were used for the primary inferential analysis.

An exploratory receiver operating characteristic (ROC) analysis was performed among patients who survived to 90 days and had an available 90-day MoCA assessment to evaluate the ability of daily energy intake (kcal/kg/day) to discriminate a 90-day MoCA score ≥ 22. Discrimination was quantified using the area under the ROC curve (AUC), and the 95% CI for the AUC was estimated using 2000 stratified bootstrap replicates. The optimal within-sample threshold was identified using Youden’s index. Because the cognitive threshold, energy-intake cut-off, and discriminatory performance were evaluated within the same cohort, the ROC analysis was considered exploratory and requires external validation. Complete-case analysis was used for each statistical model, and missing values were not imputed.

## 3. Results

### 3.1. Study Cohort and Baseline Characteristics

A total of 100 patients were included in the overall study cohort, comprising 50 patients in the lower energy-intake group and 50 patients in the higher energy-intake group. At 90 days, 12 patients had died (mRS 6), including 9 patients in the lower energy-intake group and 3 patients in the higher energy-intake group. These patients remained included in the overall functional-outcome and mortality analyses but did not contribute to analyses requiring a 90-day cognitive assessment. Consequently, the 90-day cognitive-analysis population comprised 88 survivors: 41 in the lower energy-intake group and 47 in the higher energy-intake group.

The mean age was 67.5 ± 14.1 years in the low energy-intake group and 72.1 ± 11.0 years in the higher energy-intake group (*p* = 0.072). Men represented 56.0% of each group. No significant between-group differences were observed in BMI, body weight, hypertension, dyslipidaemia, or serum albumin.

Atrial fibrillation was more frequent in the low energy-intake group than in the higher energy-intake group (44.0% vs. 10.0%, *p* < 0.001). Chronic coronary artery disease was also more frequent in the low energy-intake group (58.0% vs. 34.0%, *p* = 0.016). Conversely, median CRP was higher in the higher energy-intake group than in the low energy-intake group (52.5 [26.2–138.0] vs. 11.0 [4.0–116.8] mg/dL, *p* = 0.004).

ACE inhibitor use was more frequent in the low energy-intake group (32.0% vs. 14.0%, *p* = 0.032). No significant differences were observed in calcium-channel blocker, beta-blocker, antiplatelet, statin, or antibiotic use. The complete baseline clinical and treatment characteristics are presented in Table 1.

### 3.2. Imaging and Occlusion Characteristics

CT was the imaging modality in 49 patients (98.0%) in the low energy-intake group and 19 patients (38.0%) in the higher energy-intake group (*p* < 0.001).

The proportions of tandem occlusion, ICA terminus occlusion, M1 occlusion, M2 inferior division occlusion, M2 superior division occlusion, and basilar artery occlusion did not differ significantly between groups.

PICA/AICA/SCA occlusion showed a marked between-group imbalance, being present in none of the patients in the low energy-intake group and in 38 patients (76.0%) in the higher energy-intake group (*p* < 0.001) (Table 1).

### 3.3. Nutritional Intake

Mean daily energy intake was significantly higher in the higher energy-intake group than in the low energy-intake group, both in absolute terms and after normalization to body weight.

Absolute daily energy intake was 2541.6 ± 332.3 kcal/day in the higher energy-intake group and 2181.8 ± 459.1 kcal/day in the low energy-intake group (*p* < 0.001). Energy intake relative to body weight was 35.5 ± 3.4 kcal/kg/day and 29.1 ± 0.7 kcal/kg/day, respectively (*p* < 0.001).

Daily protein intake was significantly greater in the higher energy-intake group (117.9 ± 20.8 vs. 91.1 ± 19.4 g/day, *p* < 0.001), as was daily lipid intake (94.0 ± 16.7 vs. 73.7 ± 15.6 g/day, *p* < 0.001). Daily carbohydrate intake did not differ significantly between groups (305.9 ± 47.5 vs. 288.5 ± 62.9 g/day, *p* = 0.120).

### 3.4. Neurological and Functional Outcomes

Stroke severity at admission was comparable between groups. Median admission NIHSS was 13.5 [11.0–15.0] in the low energy-intake group and 13.0 [11.2–14.8] in the higher energy-intake group (*p* = 0.986).

There were no statistically significant differences in NIHSS at discharge (5.0 [4.0–8.0] vs. 5.0 [4.0–5.8], *p* = 0.108) or at 90 days (3.0 [2.0–3.0] vs. 2.5 [2.0–3.0], *p* = 0.674).

**Figure 1:**
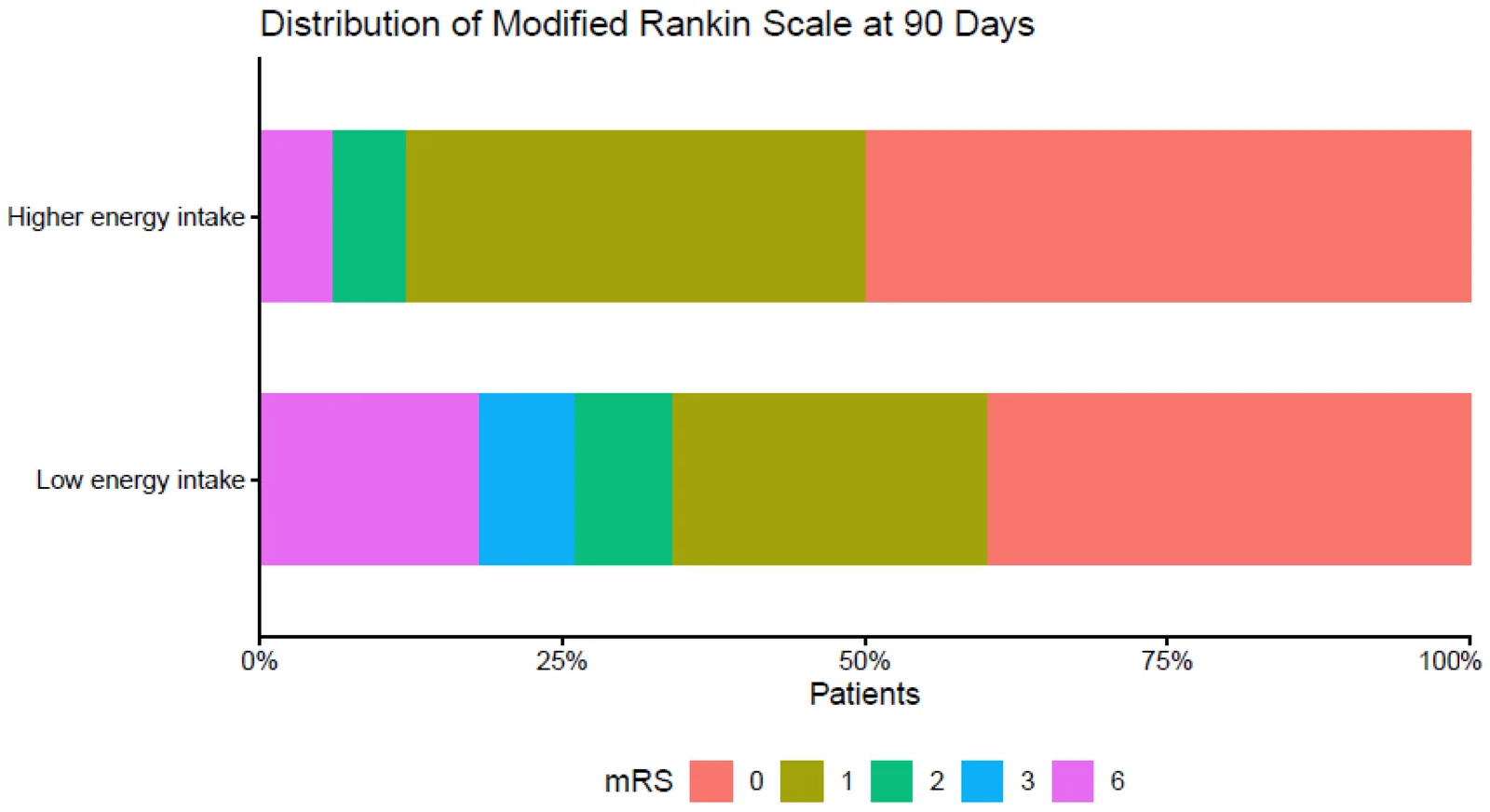
Distribution of the Modified Rankin Scale (mRS) scores at 90 days according to daily energy intake group. Stacked bar chart showing the distribution of functional outcomes at 90 days in patients with lower (<31 kcal/kg/day) and higher (≥31 kcal/kg/day) daily energy intake. Each color represents one mRS category, and bar lengths indicate the proportion of patients within each group.

Pre-stroke mRS > 0 was present in 11 patients (22.0%) in the low energy-intake group and 12 patients (24.0%) in the higher energy-intake group (*p* = 0.812). At 90 days, mRS > 0 was present in 30 patients (60.0%) and 25 patients (50.0%), respectively (*p* = 0.315). The full 90-day mRS distribution showed overlap between groups, although deaths, represented by mRS 6, were numerically more frequent in the low energy-intake group (Figure 1).

Nine patients (18.0%) in the low energy-intake group and three patients (6.0%) in the higher energy-intake group had an mRS score of 6 at 90 days. This difference did not reach statistical significance in an exploratory Fisher’s exact test (*p* = 0.121).

### 3.5. Cognitive Outcomes

Cognitive outcome analyses at 90 days were restricted to the 88 surviving patients with available MoCA assessments (41 in the lower energy-intake group and 47 in the higher energy-intake group). Baseline MoCA was modestly higher in the higher energy-intake group than in the lower energy-intake group (20.4 ± 2.2 vs. 19.5 ± 1.3, *p* = 0.026).

At 90 days, a larger between-group difference in MoCA score was observed. Mean 90-day MoCA was 24.2 ± 2.1 in the higher energy-intake group and 20.0 ± 1.5 in the low energy-intake group (*p* < 0.001) (Table 1).

**Figure 2:**
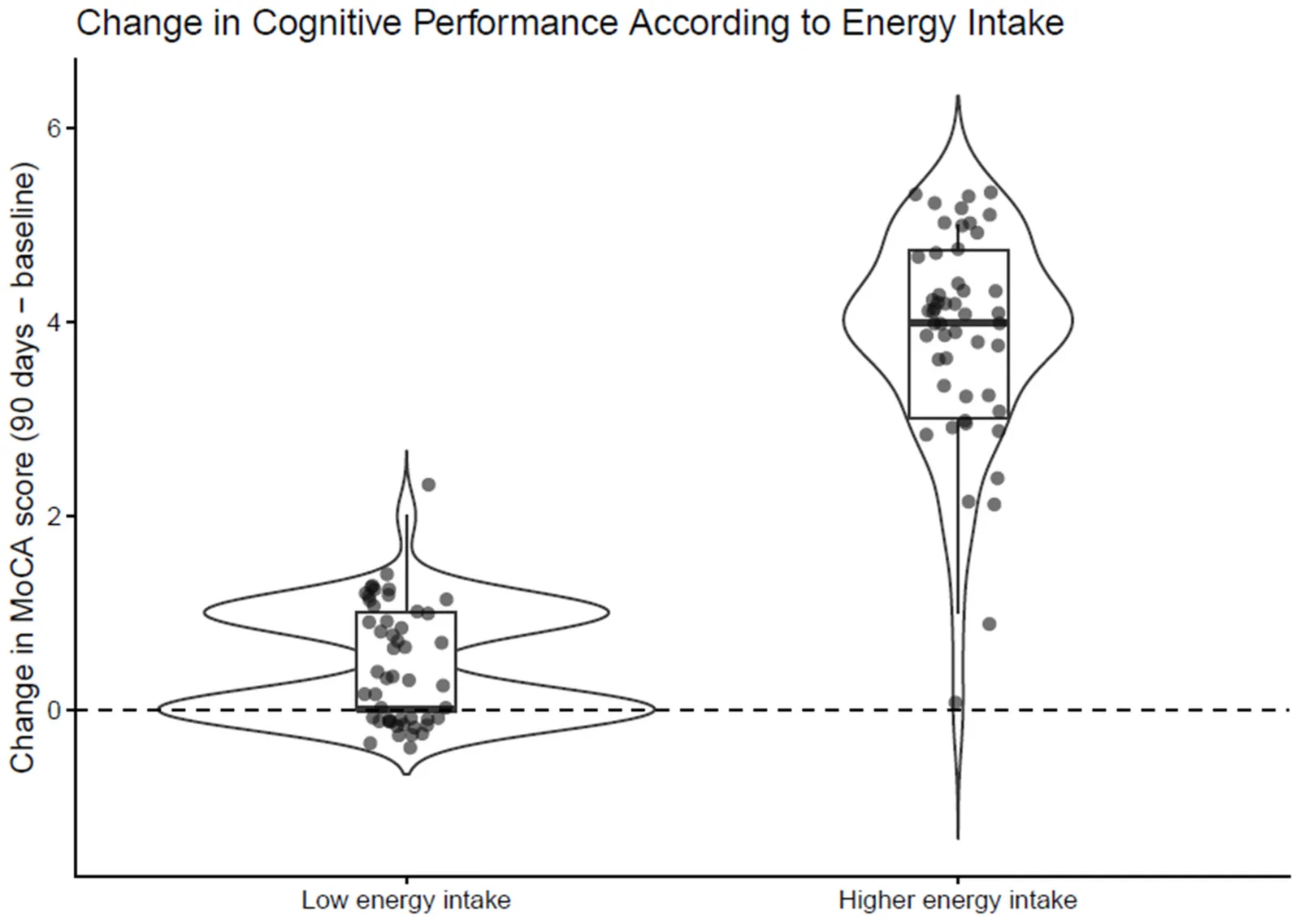
Change in Montreal Cognitive Assessment (ΔMoCA) score according to daily energy intake group. Violin plots with overlaid boxplots and individual observations show the distribution of cognitive change from baseline to 90 days in the lower and higher energy-intake groups. The dashed horizontal line represents no cognitive change (ΔMoCA = 0).

Mean ΔMoCA was 3.82 ± 1.08 points in the higher energy-intake group and 0.48 ± 0.54 points in the low energy-intake group. The absolute between-group difference in mean cognitive improvement was therefore 3.34 points (*p* < 0.001). The distribution of ΔMoCA differed between the energy-intake groups, with higher values observed in the higher energy-intake group (Figure 2).

### 3.6. Multivariable Analysis of 90-Day MoCA

Daily energy intake normalized to body weight was evaluated as a continuous exposure in the primary multivariable model. Among the 88 survivors with available 90-day cognitive assessment, each additional 1 kcal/kg/day was associated with a 0.18-point higher 90-day MoCA score after adjustment for baseline MoCA, atrial fibrillation, chronic coronary artery disease, admission NIHSS, CRP, and PICA/AICA/SCA occlusion (adjusted β = 0.179, HC3 95% CI 0.081–0.278; *p* < 0.001).

Baseline MoCA was positively associated with 90-day MoCA (β = 0.899, 95% CI 0.666–1.131; *p* < 0.001). CRP showed a small positive association (β = 0.0037, 95% CI 0.0002–0.0071; *p* = 0.041), and PICA/AICA/SCA occlusion was also associated with higher 90-day MoCA (β = 1.824, 95% CI 0.946–2.702; *p* < 0.001). Atrial fibrillation, chronic coronary artery disease, and admission NIHSS were not independently associated with 90-day MoCA in this model (Table 2).

As an additional assessment of estimate stability, 2000 bootstrap replicates were performed for the continuous energy-intake coefficient. The bootstrap 95% CI was 0.081–0.266, consistent with the HC3-based inference. These estimates represent adjusted associations and should not be interpreted as causal effects.

**Figure 3:**
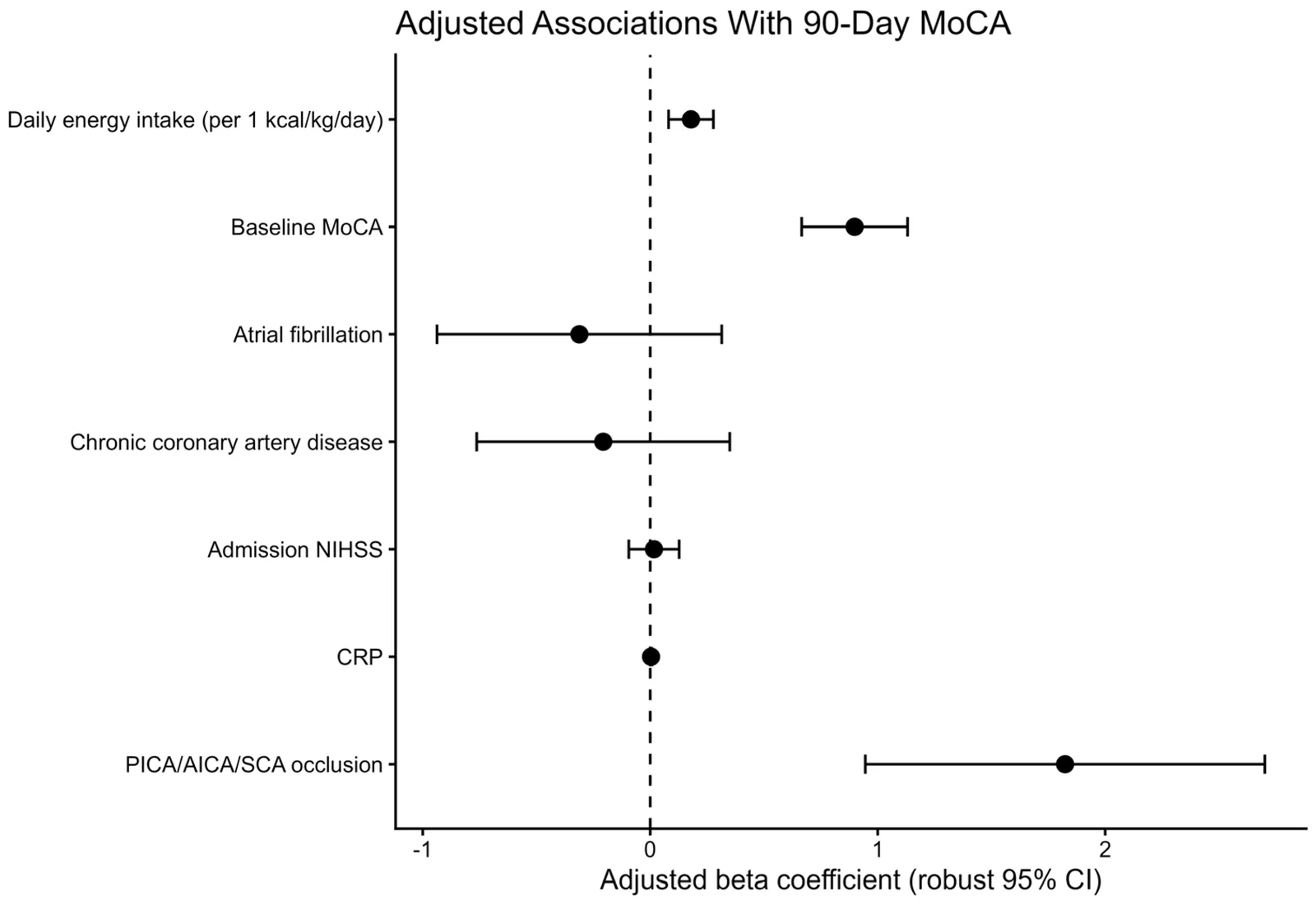
Adjusted associations with 90-day Montreal Cognitive Assessment (MoCA) score. Forest plot showing adjusted β coefficients and HC3 95% confidence intervals from the primary multivariable linear regression model in which daily energy intake was analyzed continuously in kcal/kg/day. The model was adjusted for baseline MoCA, atrial fibrillation, chronic coronary artery disease, admission NIHSS, CRP, and PICA/AICA/SCA occlusion. The vertical dashed line represents the null value (β = 0). Estimates represent associations rather than causal effects.

The overall model was statistically significant (*p* < 0.001) and included patients with complete 90-day cognitive data. The adjusted coefficients and confidence intervals are shown in the forest plot (Figure 3).

### 3.7. Exploratory Categorical and Sensitivity Analyses

The internally derived 31 kcal/kg/day threshold was retained for exploratory categorical analyses. After adjustment for baseline MoCA, atrial fibrillation, chronic coronary artery disease, admission NIHSS, CRP, and PICA/AICA/SCA occlusion, membership in the higher energy-intake group remained associated with a higher 90-day MoCA score (adjusted β = 3.23, HC3 95% CI 2.23–4.23; *p* < 0.001). Because the 31 kcal/kg/day categorization was derived from the same cohort, this estimate should be interpreted as exploratory and not as evidence of a clinical treatment threshold.

In an expanded continuous-exposure sensitivity model additionally incorporating age, sex, and pre-stroke mRS, the association between daily energy intake and 90-day MoCA remained similar (β = 0.176 per 1 kcal/kg/day, HC3 95% CI 0.071–0.281; *p* = 0.002). This analysis addresses additional available indicators of baseline clinical status but cannot account for unmeasured determinants such as swallowing function, rehabilitation intensity, mood, education, or premorbid cognitive reserve.

Because PICA/AICA/SCA occlusion showed the most pronounced between-group imbalance, an additional sensitivity analysis excluded patients with PICA/AICA/SCA occlusion. Within this restricted population, continuous daily energy intake remained associated with 90-day MoCA (β = 0.292 per 1 kcal/kg/day, HC3 95% CI 0.134–0.451; *p* < 0.001). This analysis reduces the influence of the markedly imbalanced occlusion category but does not eliminate residual confounding.

**Figure 4:**
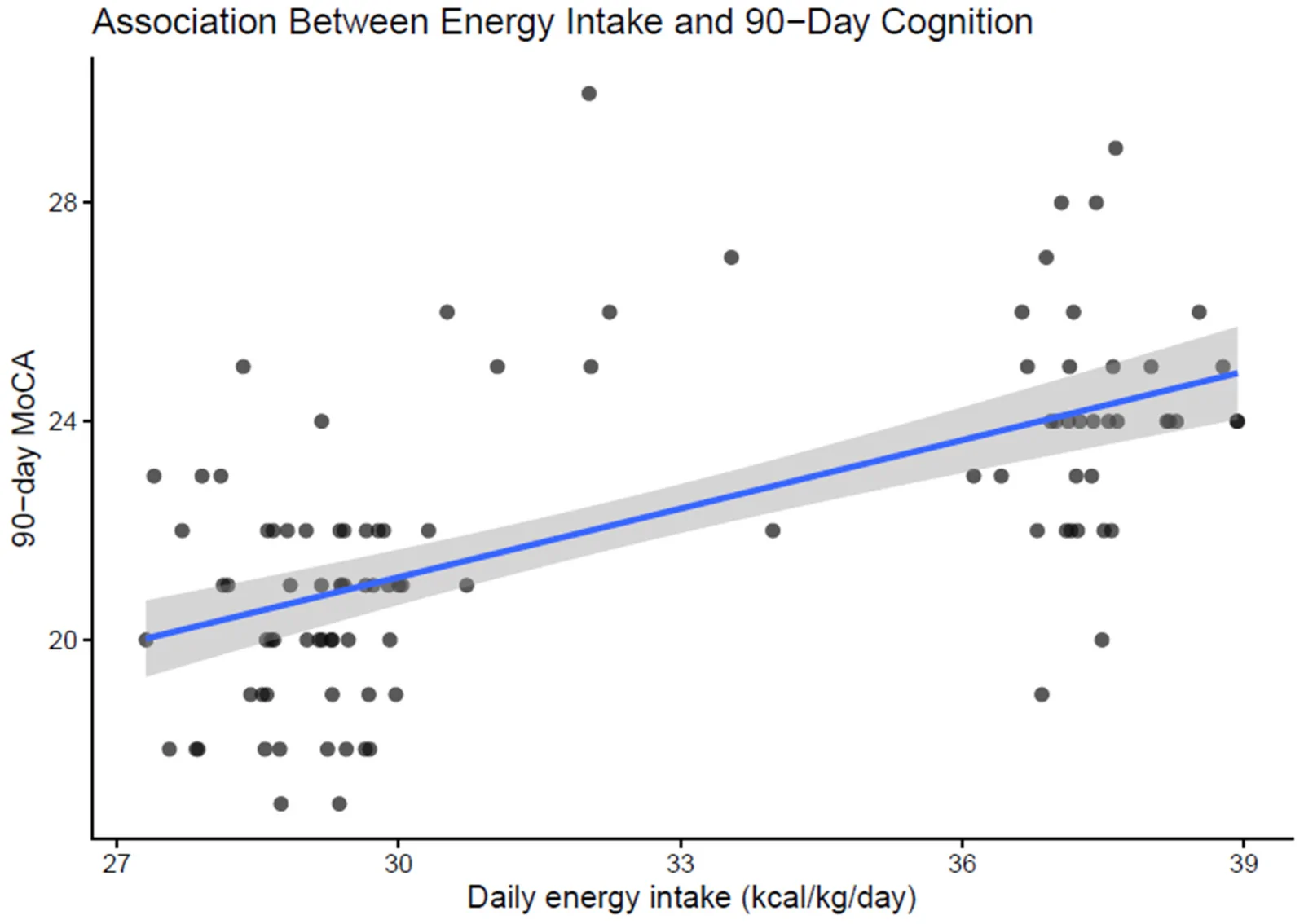
Association between daily energy intake (kcal/kg/day) and 90-day Montreal Cognitive Assessment (MoCA) score. Each point represents an individual patient. The fitted line and 95% confidence interval provide a descriptive visualization of the relationship between daily energy intake and 90-day cognitive performance. Statistical inference was based on the adjusted multivariable regression model.

**Figure 5:**
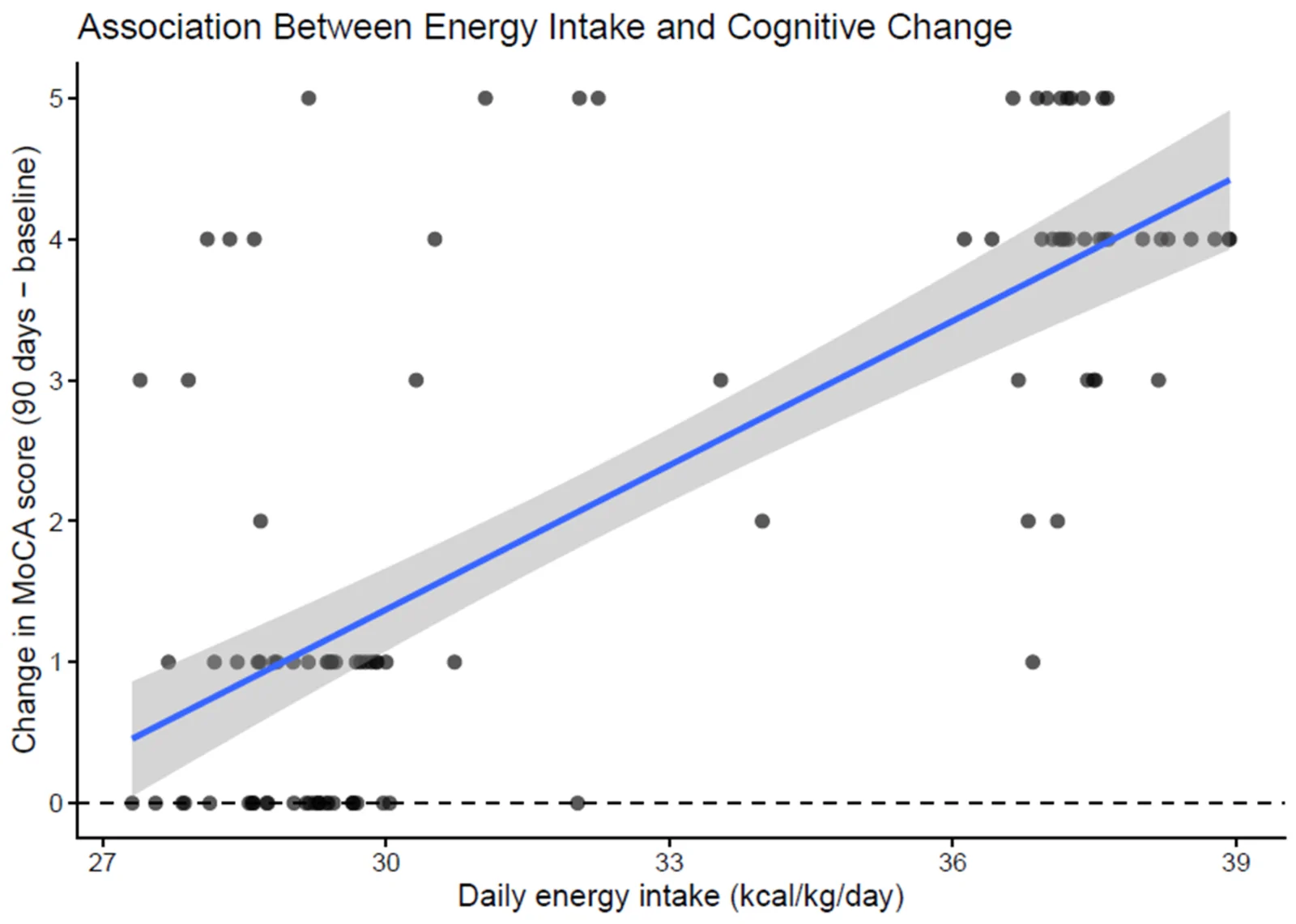
Association between daily energy intake (kcal/kg/day) and change in Montreal Cognitive Assessment (ΔMoCA) score from baseline to 90 days. Each point represents an individual patient. ΔMoCA was calculated as the 90-day MoCA score minus the baseline MoCA score. The horizontal dashed line represents no change in cognitive performance (ΔMoCA = 0). The fitted line and 95% confidence interval provide a descriptive visualization of the observed relationship; statistical inference was based on the adjusted multivariable regression analysis.

Figure 4 and Figure 5 provide descriptive visualizations of the observed relationships between daily energy intake and 90-day MoCA and ΔMoCA, respectively. Statistical inference was based on the adjusted regression analyses.

These continuous-exposure analyses support an association across the observed energy-intake range but should not be interpreted as establishing a causal dose–response effect.

### 3.8. Propensity-Score Sensitivity Analysis

Propensity-score matching was performed as a sensitivity analysis of the exploratory categorical exposure groups. However, conventional matching did not achieve adequate covariate balance. After matching, substantial residual imbalance remained for several variables, including atrial fibrillation (absolute SMD = 1.195), PICA/AICA/SCA occlusion (absolute SMD = 0.847), admission NIHSS (absolute SMD = 0.523), BMI (absolute SMD = 0.513), hypertension (absolute SMD = 0.343), sex (absolute SMD = 0.337), and pre-stroke mRS (absolute SMD = 0.314). Accordingly, the conventional propensity-score matched sample was not considered sufficiently balanced for a reliable causal interpretation.

Because PICA/AICA/SCA occlusion was completely absent in the lower-intake group but common in the higher-intake group, an additional analysis imposed exact matching on PICA/AICA/SCA status. Although this eliminated imbalance in that variable, substantial imbalance persisted for other covariates, including atrial fibrillation (absolute SMD = 0.896), BMI (0.670), chronic coronary artery disease (0.528), dyslipidaemia (0.509), sex (0.506), pre-stroke mRS (0.471), and baseline MoCA (0.449). Therefore, exact matching did not resolve the broader lack of comparability between the exploratory exposure groups.

These findings indicate limited propensity-score overlap between the exploratory energy-intake groups and reinforce the use of continuous energy intake as the principal exposure, while emphasizing that residual confounding cannot be eliminated in this observational cohort.

### 3.9. Exploratory ROC Analysis

**Figure 6:**
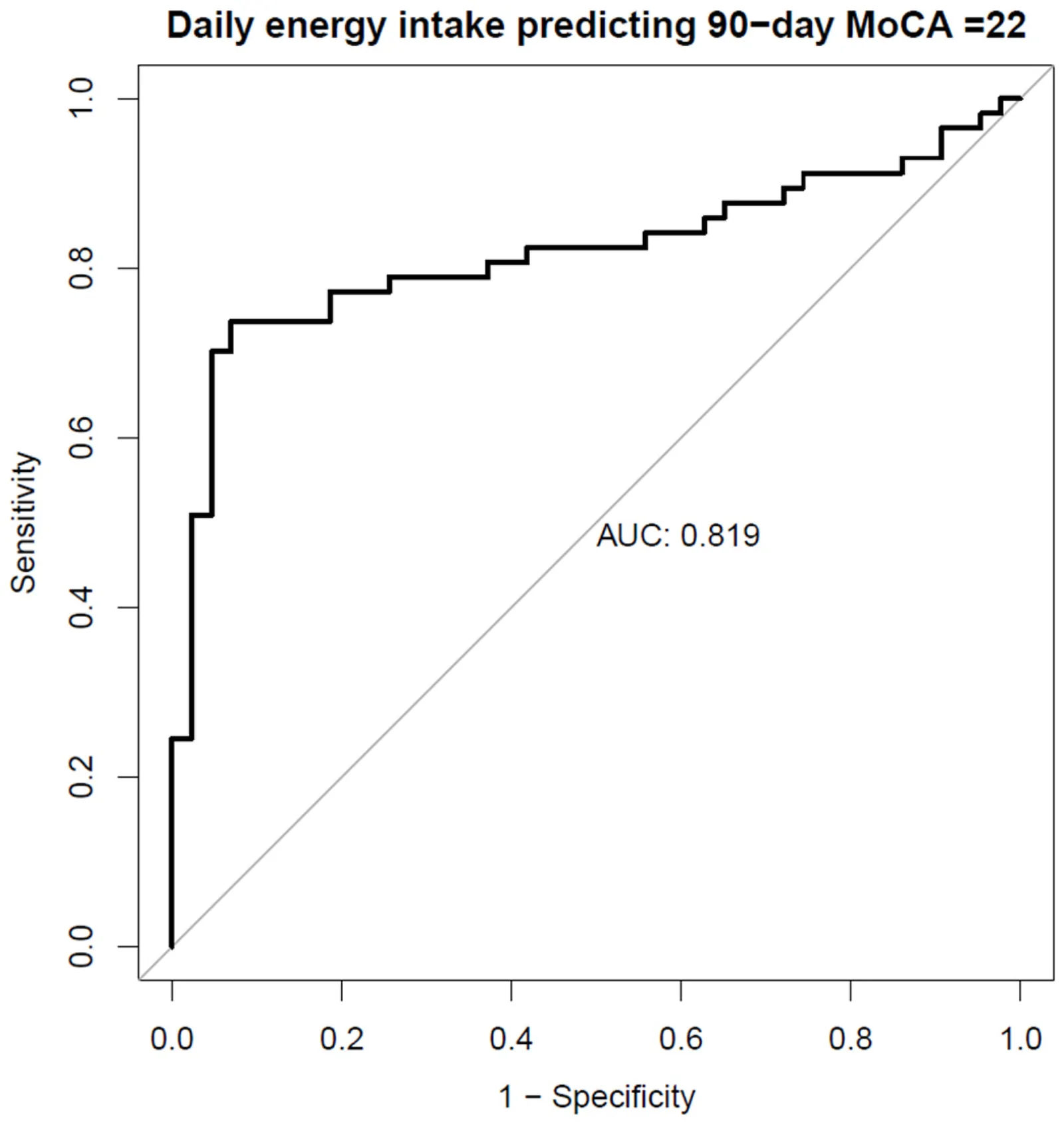
Exploratory receiver operating characteristic (ROC) analysis of daily energy intake (kcal/kg/day) for discriminating a 90-day Montreal Cognitive Assessment (MoCA) score ≥ 22 among survivors with available 90-day cognitive assessment (*n* = 88). The area under the curve (AUC) was 0.819 (bootstrap 95% CI 0.710–0.904). The within-sample threshold identified by Youden’s index was 30.18 kcal/kg/day, with a sensitivity of 78.0% and specificity of 92.1%. The analysis was internally derived and has not been externally validated.

Among the 88 survivors with available 90-day cognitive assessment, exploratory ROC analysis of daily energy intake for distinguishing a 90-day MoCA score ≥22 yielded an AUC of 0.819 (bootstrap 95% CI 0.710–0.904) (Figure 6). The within-sample optimal threshold identified by Youden’s index was 30.18 kcal/kg/day, with 78.0% sensitivity and 92.1% specificity. The 31 kcal/kg/day categorization used in the exploratory group analyses was retained from the original categorical analysis and was not redefined using the revised survivor-based ROC analysis. Because both the threshold and discriminatory performance were estimated within the same cohort, these findings should not be interpreted as a validated clinical cut-off and require external validation.

The ROC analysis was intended solely as an exploratory assessment of discrimination within the present cohort. Because the AUC and optimal threshold were estimated in the same sample, their performance may be optimistic. The difference between the ROC-derived threshold of 30.18 kcal/kg/day and the 31 kcal/kg/day threshold used in the exploratory categorical analyses further supports avoiding interpretation of either value as a definitive clinical cut-off. External validation in an independent cohort is required before any energy-intake threshold can be considered for clinical application.

## 4. Discussion

The principal finding of this multicenter retrospective observational study was an association between higher daily energy intake normalized to body weight and higher cognitive performance at 90 days among survivors of ischemic stroke treated with mechanical thrombectomy. In the primary analysis, treating energy intake as a continuous exposure, each additional 1 kcal/kg/day was associated with a 0.18-point higher 90-day MoCA score after adjustment for baseline MoCA, atrial fibrillation, chronic coronary artery disease, admission NIHSS, CRP, and PICA/AICA/SCA occlusion (β = 0.179, HC3 95% CI 0.081–0.278; *p* < 0.001). A 2000-replicate bootstrap analysis yielded a similar estimate and confidence interval (β = 0.179; bootstrap 95% CI 0.081–0.266), supporting the stability of the continuous-exposure association within this cohort.

The association was also observed in additional sensitivity analyses. In an expanded model incorporating available indicators of baseline clinical status, the estimate was similar (β = 0.176, HC3 95% CI 0.071–0.281; *p* = 0.002). After excluding patients with PICA/AICA/SCA occlusion, each additional 1 kcal/kg/day remained associated with a 0.29-point higher 90-day MoCA score (β = 0.292, HC3 95% CI 0.134–0.451; *p* < 0.001). Exploratory analysis using the internally derived categorical grouping also demonstrated an association between higher-intake group membership and higher 90-day MoCA (β = 3.23, HC3 95% CI 2.23–4.23; *p* < 0.001). These analyses demonstrate consistency of the observed association across several model specifications but do not establish a causal effect of energy intake on cognitive recovery.

### 4.1. Principal Findings

Post-stroke recovery is metabolically demanding and involves neuronal reorganization, synaptic plasticity, tissue repair, inflammatory regulation, maintenance of muscle mass, and active participation in rehabilitation. Insufficient nutritional intake could plausibly influence these processes through negative energy balance, fatigue, loss of lean body mass, and reduced rehabilitation tolerance; however, these mechanisms cannot be established from the present observational data.

Patients in the higher energy-intake group consumed not only more total energy but also more protein and lipids. Consequently, the observed cognitive association cannot be attributed to energy intake alone. Greater protein availability may support muscle preservation and physical reserve, whereas lipid intake may contribute to cellular membrane structure and neuronal metabolism. The exposure in the present study should therefore be interpreted as a broader nutritional pattern characterized by greater energy, protein, and lipid intake.

The between-group cognitive differences observed in the exploratory categorical analysis are directionally consistent with previous evidence examining nutritional support during stroke rehabilitation. Aquilani et al. [15] reported greater MMSE improvement among patients receiving calorie and protein supplementation than among controls. In the present cohort, mean ΔMoCA was 3.82 points in the higher energy-intake group and 0.48 points in the lower energy-intake group. However, because group allocation was observational and the 31 kcal/kg/day categorization was internally derived, this comparison should be interpreted as descriptive and hypothesis-generating rather than as evidence of a treatment effect.

Direct numerical comparison between the studies should be avoided because they used different cognitive instruments, follow-up durations, study designs, and nutritional exposures. Nevertheless, the direction of the findings is similar and supports further prospective investigation [17,18,19,20].

### 4.2. Baseline Cognition and the Adjusted Association

Baseline MoCA was modestly but significantly higher in the higher energy-intake group. Adjustment for baseline cognition was therefore essential to reduce bias from differences in initial cognitive status.

Baseline MoCA was strongly associated with follow-up cognition. In the primary continuous-exposure model, each additional baseline MoCA point was associated with an approximately 0.90-point higher 90-day MoCA score (β = 0.899, HC3 95% CI 0.666–1.131; *p* < 0.001). Importantly, continuous daily energy intake remained associated with 90-day MoCA after adjustment for baseline cognition and the other prespecified measured covariates.

In the exploratory categorical comparison, the between-group difference increased from 0.82 MoCA points at baseline to 4.16 points at 90 days, and ΔMoCA also differed between groups. Although these observations indicate that the 90-day difference was not explained solely by the measured baseline MoCA difference, they cannot exclude confounding by other baseline characteristics or reverse causality.

However, adjustment for baseline MoCA does not account for all determinants of cognitive reserve. Education, premorbid cognitive status, lesion burden, depression, delirium, rehabilitation intensity, and social factors were not fully characterized and may have influenced recovery.

### 4.3. Possible Biological Mechanisms

Several biological mechanisms could plausibly contribute to the observed association between daily energy intake and 90-day cognitive performance after acute ischemic stroke. The brain has high energy demands, and these increase further after ischemic injury because of the metabolic processes involved in tissue repair and functional recovery. From a biological perspective, adequate energy availability could support metabolic processes involved in neuronal repair and synaptic remodeling after ischemic injury; however, the present study cannot determine whether these mechanisms explain the observed association [26,27].

These mechanisms provide biological plausibility for the observed association but should not be interpreted as evidence of a causal mechanism in the present cohort. Experimental stroke-recovery literature has identified synaptogenesis, axonal sprouting, and cortical reorganization as important components of neurological recovery [28,29]. Although adequate substrate availability is biologically plausible during these energy-demanding processes, the present study cannot determine whether nutritional intake directly modifies these mechanisms. Another possible explanation is the role of macronutrients. Patients with higher energy intake in our study also consumed greater amounts of protein and lipids. Protein supplies amino acids required for tissue repair and neurotransmitter synthesis, while lipids are important components of neuronal membranes and myelin. Although our study was not designed to evaluate the independent effects of individual macronutrients, their combined contribution may partly characterize the broader nutritional pattern associated with cognitive outcome in this cohort. Inflammation may also influence recovery. Acute ischemic stroke triggers a systemic inflammatory response that can contribute to secondary brain injury and impair neurological recovery [30,31]. Adequate daily energy intake may help reduce the effects of this catabolic state by preserving nutritional reserves and supporting normal immune function. Although inflammation is biologically relevant to post-stroke recovery, in the primary multivariable model, CRP showed a small positive association with 90-day MoCA (β = 0.0037, HC3 95% CI 0.0002–0.0071; *p* = 0.041). Given the small coefficient, observational design, and multiple correlated clinical characteristics, this finding should be interpreted cautiously and does not establish a mechanistic relationship between systemic inflammation and cognitive outcome. Finally, patients with better nutritional status may be able to participate more actively in rehabilitation. Adequate nutritional intake may support physical reserve and rehabilitation participation; however, rehabilitation intensity was not systematically available in the present cohort and may itself have confounded the observed association [23,32].

Although these mechanisms are biologically plausible, the observational design of our study does not allow conclusions about causality.

Prospective studies with standardized nutritional assessment and, ultimately, appropriately designed intervention studies are required to determine whether modification of nutritional intake influences cognitive recovery after mechanical thrombectomy.

### 4.4. Cognitive Versus Neurological Recovery

An important finding was the absence of significant between-group differences in NIHSS at admission, discharge, or 90 days. Similarly, the proportion of patients with mRS > 0 did not differ significantly between groups.

This apparent dissociation between cognitive and conventional neurological outcomes is clinically plausible. NIHSS primarily measures focal deficits such as motor weakness, sensory loss, visual field deficits, language impairment, and level of consciousness. It does not comprehensively assess executive function, attention, memory, or visuospatial performance [24].

The mRS is a global disability scale and may be insensitive to cognitive differences in patients who remain physically independent [25]. Patients may therefore have similar NIHSS and mRS outcomes but experience meaningfully different cognitive trajectories.

These findings support the inclusion of formal cognitive assessment as a distinct outcome in stroke recovery studies. Reliance exclusively on NIHSS and mRS may overlook cognitive outcomes that are important for medication adherence, decision-making, return to work, social reintegration, and long-term independence [2,3,4,5].

### 4.5. Functional Outcome and Mortality

The proportion of patients with mRS > 0 at 90 days was numerically lower in the higher energy-intake group, although the difference did not reach statistical significance. Similarly, the complete ordinal distribution of mRS scores showed substantial overlap between groups. These findings suggest that the observed association with cognitive outcome was not accompanied by a statistically significant between-group difference in the measured global functional outcomes [25]. This observation is consistent with previous studies demonstrating that cognitive recovery and functional independence, although related, represent distinct dimensions of post-stroke recovery and may not improve in parallel [6,10,23].

Death at 90 days, defined as mRS = 6, occurred in 18.0% of patients in the lower energy-intake group compared with 6.0% in the higher energy-intake group. Although this numerical difference may be clinically relevant, it did not reach statistical significance and should therefore be interpreted cautiously. Large randomized thrombectomy trials have consistently demonstrated that mortality is influenced by multiple factors, including baseline stroke severity, age, infarct volume, reperfusion success, and medical complications, making it unlikely that daily energy intake alone determines survival [2,3,4,5].

The mortality comparison in our study should be considered exploratory because it was based on a relatively small number of events. In addition, the observed association may be affected by reverse causation, whereby patients with more severe neurological deficits or medical complications consumed less energy as a consequence of reduced consciousness, dysphagia, systemic infection, gastrointestinal intolerance, or limitations in active treatment. Similar concerns have been raised in previous studies investigating nutritional status after stroke [16,17,18,19].

No Kaplan–Meier survival analysis or Cox proportional hazards regression was performed because the study was not designed to evaluate time-to-event outcomes and only the vital status at 90 days was available. Therefore, mortality should be interpreted as a comparison of 90-day mortality proportions rather than as a survival analysis.

### 4.6. Energy Intake Relative to Body Weight

Expressing daily energy intake in kcal/kg/day provides greater clinical interpretability than reporting kcal/day alone. The same absolute energy intake may represent relative underfeeding in a heavier patient and comparatively high intake in a lighter patient.

In the revised primary continuous-exposure analysis, each additional 1 kcal/kg/day was associated with an adjusted 0.18-point higher 90-day MoCA score (β = 0.179, HC3 95% CI 0.081–0.278). Across a 5 kcal/kg/day difference, the fitted linear model would correspond to an approximately 0.90-point difference in 90-day MoCA within the observed exposure range. This calculation describes the fitted statistical association and should not be interpreted as the expected cognitive benefit of prescribing an additional 5 kcal/kg/day. This estimate should not be extrapolated beyond the study data or interpreted as a treatment effect. Individual nutritional requirements vary according to body composition, age, metabolic stress, activity, rehabilitation intensity, organ function, and nutritional status.

Furthermore, the relatively narrow distribution of energy intake in the low-intake group and the broader distribution in the higher-intake group may influence the fitted linear relationship [20,33,34,35].

### 4.7. Interpretation of ROC Findings

In the analysis restricted to the 88 survivors with available 90-day cognitive assessment, daily energy intake demonstrated an AUC of 0.819 (bootstrap 95% CI 0.710–0.904) for discriminating a 90-day MoCA score ≥ 22. The within-sample threshold identified by Youden’s index was 30.18 kcal/kg/day, with 78.0% sensitivity and 92.1% specificity.

This analysis was exploratory and should not be interpreted as establishing a clinically actionable nutritional threshold. Both the discrimination estimate and optimal threshold were derived within the same cohort and may therefore be optimistic. Furthermore, the ROC-derived threshold of 30.18 kcal/kg/day was not identical to the 31 kcal/kg/day categorization used in the exploratory group analyses, reinforcing that no specific cut-off can be considered validated from these data. External validation in an independent cohort is required before any energy-intake threshold can be considered for clinical use.

### 4.8. Baseline Imbalances, Propensity-Score Analysis, and Residual Confounding

Substantial baseline differences were present between the exploratory energy-intake groups, particularly for atrial fibrillation, chronic coronary artery disease, imaging modality, and PICA/AICA/SCA occlusion. The complete absence of PICA/AICA/SCA occlusion in the lower-intake group and its high prevalence in the higher-intake group was particularly important and suggests that the groups represented substantially different stroke phenotypes or clinical pathways.

Propensity-score matching was performed as an additional sensitivity analysis. Conventional 1:1 matching did not achieve adequate overall covariate balance. Residual absolute standardized mean differences after matching remained substantial for several variables, including atrial fibrillation (1.195), PICA/AICA/SCA occlusion (0.847), admission NIHSS (0.523), BMI (0.513), hypertension (0.343), sex (0.337), and pre-stroke mRS (0.314). Accordingly, the propensity-score matched outcome estimate was not interpreted as a confounding-adjusted causal effect.

An additional matching analysis requiring exact balance on PICA/AICA/SCA status successfully balanced that variable but continued to show substantial imbalance in other characteristics, including atrial fibrillation, BMI, chronic coronary artery disease, sex, and baseline MoCA. Thus, neither matching strategy adequately reproduced a balanced comparison between the exploratory exposure groups.

These findings provide empirical evidence of limited comparability between the categorical exposure groups and reinforce the decision to emphasize daily energy intake as a continuous exposure rather than relying on the internally derived dichotomy. Although the continuous multivariable association remained present after adjustment and in sensitivity analyses, regression adjustment cannot eliminate residual or unmeasured confounding. The results should therefore be interpreted as adjusted observational associations rather than causal effects.

### 4.9. Clinical Implications

The present findings reinforce the relevance of assessing nutritional status and dietary intake as part of comprehensive stroke care but do not establish a therapeutic energy-intake target. Daily energy intake was associated with cognitive outcome in this cohort; however, the observational design, substantial baseline differences, limited propensity-score overlap, and potential for reverse causality preclude concluding that increasing caloric intake would improve cognition. Nutritional assessment and treatment should therefore continue to follow individualized clinical evaluation and established nutrition guidelines rather than the exploratory thresholds identified in this study [20,23,33].

Energy requirements after stroke vary considerably according to age, body composition, premorbid nutritional status, obesity, renal or cardiac disease, systemic inflammation, metabolic stress, and the risk of refeeding syndrome. Consequently, nutritional prescriptions should remain individualized and consistent with current clinical nutrition guidelines [20,33,34,35]. Future prospective studies should evaluate energy intake in relation to estimated individual energy requirements rather than absolute caloric intake alone. Additional variables, including protein intake normalized to body weight, swallowing function, route of nutritional support (oral, enteral, or parenteral), nutritional risk screening, body composition, rehabilitation intensity, infectious complications, and adherence to nutritional recommendations, should also be incorporated to better understand the relationship between nutrition and cognitive recovery after stroke [17,20,23,35].

### 4.10. Strengths and Limitations

The present study has several strengths. It included detailed demographic, vascular, imaging, neurological, functional, cognitive, biochemical, and nutritional data within a multicenter cohort. Cognitive status was assessed both at baseline and at 90 days, allowing evaluation of follow-up MoCA and within-patient cognitive change. Daily energy intake was normalized to body weight and was evaluated primarily as a continuous exposure, thereby reducing reliance on an internally derived categorical threshold. The association between continuous daily energy intake and 90-day MoCA remained present after multivariable adjustment, use of HC3 heteroscedasticity-consistent standard errors, bootstrap resampling, expansion of the model to incorporate additional available baseline characteristics, and an analysis excluding patients with PICA/AICA/SCA occlusion.

Several limitations should nevertheless be acknowledged. First, the retrospective observational design precludes causal inference and is particularly susceptible to reverse causality. Higher energy intake may reflect greater clinical stability, preserved swallowing function, better appetite, fewer complications, improved tolerance of oral or enteral feeding, or greater participation in rehabilitation rather than being a determinant of cognitive recovery. Swallowing function, feeding route, rehabilitation intensity, and post-stroke complications were not systematically available across all participating centers and therefore could not be comprehensively incorporated into the analyses. Consequently, the observed association should not be interpreted as evidence that increasing energy intake improves cognitive outcome.

Second, the study comprised a relatively small cohort of 100 patients, of whom 88 survivors had available 90-day cognitive assessment. No formal a priori sample-size calculation was performed because all consecutive eligible patients during the predefined study period were included. For the seven-predictor primary regression model, a sensitivity analysis indicated that, with 88 patients, a two-sided α of 0.05, and 80% power, the minimum detectable Cohen’s f^2^ was approximately 0.178. Thus, the available sample had limited ability to detect small associations, and the precision and generalizability of the findings remain limited. Larger independent cohorts are required for confirmation.

Third, substantial baseline differences were present between the exploratory energy-intake groups, particularly for atrial fibrillation, chronic coronary artery disease, imaging modality, baseline MoCA, and PICA/AICA/SCA occlusion. The marked imbalance in PICA/AICA/SCA occlusion suggests that the groups may have represented substantially different stroke phenotypes or clinical pathways. Propensity-score matching was performed as a sensitivity analysis; however, conventional 1:1 matching did not achieve adequate covariate balance. Important residual standardized mean differences remained for atrial fibrillation, PICA/AICA/SCA occlusion, admission NIHSS, BMI, hypertension, sex, and pre-stroke mRS. Exact matching on PICA/AICA/SCA status eliminated imbalance in that variable but resulted in persistent imbalance in several other baseline characteristics. Consequently, the propensity-score analyses do not eliminate the possibility of confounding and were not used as the principal estimates of association.

Fourth, several potentially important determinants of cognitive recovery were not systematically available, including educational attainment, detailed premorbid cognitive status, quantitative lesion burden and location, depression and other mood disorders, delirium, cognitive reserve, rehabilitation intensity, and social and environmental factors. These variables may influence both nutritional intake and cognitive recovery and therefore represent potential sources of residual confounding. Multivariable adjustment for measured baseline characteristics cannot eliminate the influence of unmeasured confounders.

Fifth, the 31 kcal/kg/day categorization used in the exploratory group analyses was internally derived and should not be interpreted as a validated clinical threshold or nutritional target. In the revised ROC analysis restricted to survivors with available 90-day cognitive assessment, the within-sample threshold identified by Youden’s index was 30.18 kcal/kg/day. The difference between these internally derived values further emphasizes the exploratory nature of threshold-based analyses. Accordingly, continuous kcal/kg/day was emphasized as the principal exposure, and external validation is required before any specific energy-intake cut-off can be considered for clinical application.

Sixth, energy, protein, and lipid intake differed simultaneously between the exploratory exposure groups and were strongly interrelated. Their independent contributions cannot therefore be separated reliably from the present data, and the observed association should not be attributed to caloric intake alone. Daily energy intake may instead partly represent a broader nutritional and clinical phenotype.

Seventh, MoCA is a validated cognitive screening instrument rather than a comprehensive neuropsychological battery. Domain-specific recovery in attention, executive function, memory, language, and visuospatial function could therefore not be evaluated in detail. MoCA performance may also be influenced by education, mood, language, and residual neurological deficits. Future studies should incorporate comprehensive domain-specific neuropsychological assessment.

Eighth, mRS was dichotomized as mRS >0 for the principal tabular between-group comparison. Although this provided a simple summary of residual disability, dichotomization results in loss of ordinal information. The complete 90-day mRS distribution was therefore additionally presented graphically.

Ninth, the number of deaths was small, and the mortality comparison was exploratory and underpowered. Cognitive outcomes at 90 days could necessarily be evaluated only among patients who survived and had an available MoCA assessment. The cognitive analyses are therefore conditional on survival and may be affected by survivor bias. These findings apply to survivors with available cognitive follow-up and should not be extrapolated to the entire thrombectomy population.

Tenth, deviations from conventional regression assumptions were identified. HC3 heteroscedasticity-consistent standard errors were therefore used for the principal inferential analysis. The continuous energy-intake association remained statistically significant using HC3 inference (β = 0.179, 95% CI 0.081–0.278; *p* < 0.001), and a 2000-replicate bootstrap analysis produced a similar confidence interval (bootstrap 95% CI 0.081–0.266). Nevertheless, these procedures address uncertainty in the fitted model and do not eliminate confounding inherent to the observational design.

Eleventh, the exploratory ROC analysis was developed and evaluated within the same cohort and may therefore overestimate discrimination in external populations. Although the AUC was 0.819 (bootstrap 95% CI 0.710–0.904), neither the AUC nor the internally derived threshold should be considered externally validated.

Finally, follow-up was limited to 90 days. Longer-term studies are required to determine whether the observed cognitive associations persist and whether they relate to quality of life, return to work, institutionalization, and long-term functional independence.

Taken together, these limitations mean that the present findings should be considered exploratory and hypothesis-generating. The analyses demonstrate an association between daily energy intake and 90-day cognitive performance among survivors after mechanical thrombectomy, but they do not establish that increasing energy intake causes cognitive improvement.

## 5. Conclusions

In this multicenter retrospective observational cohort of patients treated with mechanical thrombectomy for acute ischemic stroke, higher daily energy intake normalized to body weight was associated with higher 90-day cognitive performance among survivors with available cognitive follow-up. In the primary continuous-exposure analysis, each additional 1 kcal/kg/day was associated with a 0.18-point higher 90-day MoCA score after adjustment for measured baseline clinical and cognitive characteristics, and the association remained present across additional sensitivity analyses.

However, substantial baseline differences, inadequate covariate balance after propensity-score matching, potential residual and unmeasured confounding, reverse causality, survivor bias, the modest sample size, and the internally derived exposure thresholds preclude causal interpretation. These findings should therefore be considered exploratory and hypothesis-generating and do not establish that increasing caloric intake improves cognitive recovery. Larger prospective multicenter studies with standardized assessment of nutritional requirements, swallowing function, feeding route, rehabilitation exposure, lesion characteristics, premorbid cognitive reserve, and domain-specific cognitive outcomes are required before the clinical implications of this association can be determined.

## Figures and Tables

**Table 1:** Baseline demographic, clinical, nutritional, and outcome characteristics according to daily energy intake group.

Characteristic	Low Energy Intake (*n* = 50)	Higher Energy Intake (*n* = 50)	*p* Value
Age, years	67.5 ± 14.1	72.1 ± 11.0	0.072
Male sex, *n* (%)	28 (56.0%)	28 (56.0%)	1.000
BMI, kg/m^2^	25.9 ± 3.7	24.8 ± 4.0	0.166
Weight, kg	74.9 ± 15.1	72.7 ± 13.4	0.433
Hypertension, *n* (%)	28 (56.0%)	32 (64.0%)	0.414
Atrial fibrillation, *n* (%)	22 (44.0%)	5 (10.0%)	<0.001
Dyslipidaemia, *n* (%)	26 (52.0%)	20 (40.0%)	0.229
Chronic coronary artery disease, *n* (%)	29 (58.0%)	17 (34.0%)	0.016
Serum albumin	35.9 ± 6.0	36.9 ± 5.3	0.372
CRP, mg/dL	11.0 [4.0–116.8]	52.5 [26.2–138.0]	0.004
ACE inhibitor use, *n* (%)	16 (32.0%)	7 (14.0%)	0.032
Calcium-channel blocker use, *n* (%)	20 (40.0%)	14 (28.0%)	0.205
Beta-blocker use, *n* (%)	19 (38.0%)	21 (42.0%)	0.683
Antiplatelet use, *n* (%)	28 (56.0%)	29 (58.0%)	0.840
Statin use, *n* (%)	50 (100.0%)	50 (100.0%)	1.000
Antibiotic use, *n* (%)	11 (22.0%)	18 (36.0%)	0.123
CT imaging modality, *n* (%)	49 (98.0%)	19 (38.0%)	<0.001
Tandem occlusion, *n* (%)	1 (2.0%)	2 (4.0%)	1.000
ICA terminus occlusion, *n* (%)	4 (8.0%)	1 (2.0%)	0.362
M1 occlusion, *n* (%)	25 (50.0%)	24 (48.0%)	0.841
M2 inferior division occlusion, *n* (%)	8 (16.0%)	8 (16.0%)	1.000
M2 superior division occlusion, *n* (%)	7 (14.0%)	8 (16.0%)	0.779
Basilar artery occlusion, *n* (%)	5 (10.0%)	3 (6.0%)	0.715
PICA/AICA/SCA occlusion, *n* (%)	0 (0.0%)	38 (76.0%)	<0.001
NIHSS at admission	13.5 [11.0–15.0]	13.0 [11.2–14.8]	0.986
NIHSS at discharge	5.0 [4.0–8.0]	5.0 [4.0–5.8]	0.108
NIHSS at 90 days	3.0 [2.0–3.0]	2.5 [2.0–3.0]	0.674
Pre-stroke mRS > 0, *n* (%)	11 (22.0%)	12 (24.0%)	0.812
90-day mRS > 0, *n* (%)	30 (60.0%)	25 (50.0%)	0.315
Baseline MoCA	19.5 ± 1.3	20.4 ± 2.2	0.026
90-day MoCA	20.0 ± 1.5	24.2 ± 2.1	<0.001
Daily energy intake, kcal/day	2181.8 ± 459.1	2541.6 ± 332.3	<0.001
Daily energy intake, kcal/kg/day	29.1 ± 0.7	35.5 ± 3.4	<0.001
Daily protein intake, g/day	91.1 ± 19.4	117.9 ± 20.8	<0.001
Daily carbohydrate intake, g/day	288.5 ± 62.9	305.9 ± 47.5	0.120
Daily lipid intake, g/day	73.7 ± 15.6	94.0 ± 16.7	<0.001

Continuous variables are presented as mean ± SD; NIHSS and CRP as median [IQR]; categorical variables, including mRS > 0, as *n* (%). *p*-values are unadjusted between-group comparisons. Welch’s *t*-test was used for continuous variables, the Wilcoxon rank-sum test for NIHSS and CRP, and Pearson’s chi-square test or Fisher’s exact test for categorical variables.

**Table 2:** Multivariable linear regression analysis of factors associated with 90-day Montreal Cognitive Assessment score, with daily energy intake modeled as a continuous exposure.

Predictor	Adjusted β	HC3 95% CI	*p* Value
Daily energy intake, per 1 kcal/kg/day	0.179	0.081 to 0.278	<0.001
Baseline MoCA, per point	0.899	0.666 to 1.131	<0.001
Atrial fibrillation	−0.311	−0.937 to 0.314	0.332
Chronic coronary artery disease	−0.207	−0.763 to 0.350	0.469
NIHSS at admission, per point	0.017	−0.094 to 0.127	0.770
CRP, mg/dL	0.0037	0.0002 to 0.0071	0.041
PICA/AICA/SCA occlusion	1.824	0.946 to 2.702	<0.001

β coefficients represent adjusted differences in 90-day MoCA. Daily energy intake was modeled continuously in kcal/kg/day. Confidence intervals and *p*-values were calculated using HC3 heteroscedasticity-consistent standard errors. PICA, posterior inferior cerebellar artery; AICA, anterior inferior cerebellar artery; SCA, superior cerebellar artery; CRP, C-reactive protein; NIHSS, National Institutes of Health Stroke Scale.

## Data Availability

The datasets analyzed during the current study are available from the corresponding author upon reasonable request.

## Abbreviations

The following abbreviations are used in this manuscript:

ACE	Angiotensin-converting enzyme
AICA	Anterior inferior cerebellar artery
AUC	Area under the receiver operating characteristic curve
BMI	Body Mass Index
CI	Confidence interval
CRP	C-reactive protein
CT	Computed tomography
HC3	Heteroscedasticity-consistent type 3
ICA	Internal carotid artery
IQR	Interquartile range
MMSE	Mini-Mental State Examination
MoCA	Montreal Cognitive Assessment
MRI	Magnetic resonance imaging
mRS	Modified Rankin Scale
NHS	National Health Service
NIHSS	National Institutes of Health Stroke Scale
PICA	Posterior inferior cerebellar artery
ROC	Receiver operating characteristic
SCA	Superior cerebellar artery
SD	Standard deviation
UK	United Kingdom
